# Comparing long‐term outcomes of septal myectomy and mitral valve replacement in hypertrophic cardiomyopathy patients: A retrospective cohort study in Iran

**DOI:** 10.1002/hsr2.2045

**Published:** 2024-04-15

**Authors:** Aryan Ayati, Mehran Khoshfetrat, Saeed Davoodi, Seyed Hossein Ahmadi Tafti, Reza Arefizadeh

**Affiliations:** ^1^ Trauma and Surgery Research Center Aja University of Medical Sciences Tehran Iran; ^2^ Tehran Heart Center, Cardiovascular Diseases Research Institute Tehran University of Medical Sciences Tehran Iran

**Keywords:** cardiomyopathy, hypertrophic, mitral valve replacement, myectomy

## Abstract

**Background:**

Hypertrophic cardiomyopathy (HCM) affects millions of individuals worldwide. In severe cases, it can cause life‐threatening conditions such as left ventricular outflow tract (LVOT) obstruction, mitral regurgitation (MR), and sudden cardiac death, making surgical treatment necessary. This study aimed to report the long‐term outcomes of HCM patients undergoing septal myectomy or mitral valve replacement (MVR) and compare the results between different types of surgeries.

**Methods:**

This was a retrospective cohort study on HCM patients who underwent surgical treatment in an Iranian referral center between 2005 and 2021. Patients were divided into three groups according to the type of surgery received: septal myectomy, MVR, or a combination of both surgeries. Patient characteristics, surgical and echocardiographic features, and in‐hospital and long‐term outcomes were reported and compared between the three groups.

**Results:**

A total of 102 patients with an average age of 53.3 ± 16.9 were included. Twenty‐six patients had septal myectomy, 23 had MVR, and 53 had combined septal myectomy and MVR surgery. All surgeries were associated with a significant reduction in interventricular septum thickness and LVOT gradients. After a median of 6.8‐year follow‐up time, patients with an isolated septal myectomy had significantly lower mortality and major adverse cardiac and cerebrovascular events rates than the other groups.

**Conclusion:**

Isolated septal myectomy showed better long‐term survival rates and can correct HCM‐related MR, while MVR should be preserved only for intrinsic valve defects. More extensive studies are needed to confirm these findings and achieve a comprehensive guideline on surgical treatment of HCM.

## 1 INTRODUCTION

With a worldwide prevalence of 1:200 to 1:500, it is estimated that more than 20 million individuals are affected with hypertrophic cardiomyopathy (HCM).^1^ Multiple genes have been associated with this disorder, the most common being autosomal dominant mutations in genes related to sarcomeres. The genetic abnormalities most commonly result in myofibrillar disarray, left ventricular hypertrophy, reduced cardiac compliance, and cardiac fibrosis.^2^, ^3^ These myocardial irregularities can cause varying symptoms, including reduced exercise tolerance, chest pain, dyspnea, and arrhythmia.^4^ In severe cases, which are also known as hypertrophic obstructive cardiomyopathy, the underlying hypertrophy can cause life‐threatening conditions such as left ventricular outflow tract (LVOT) obstruction and mitral regurgitation (MR).^5^, ^6^, ^7^ Moreover, HCM is reported as the most common cause of sudden cardiac death under the age of 35.^8^

**Figure 1:**
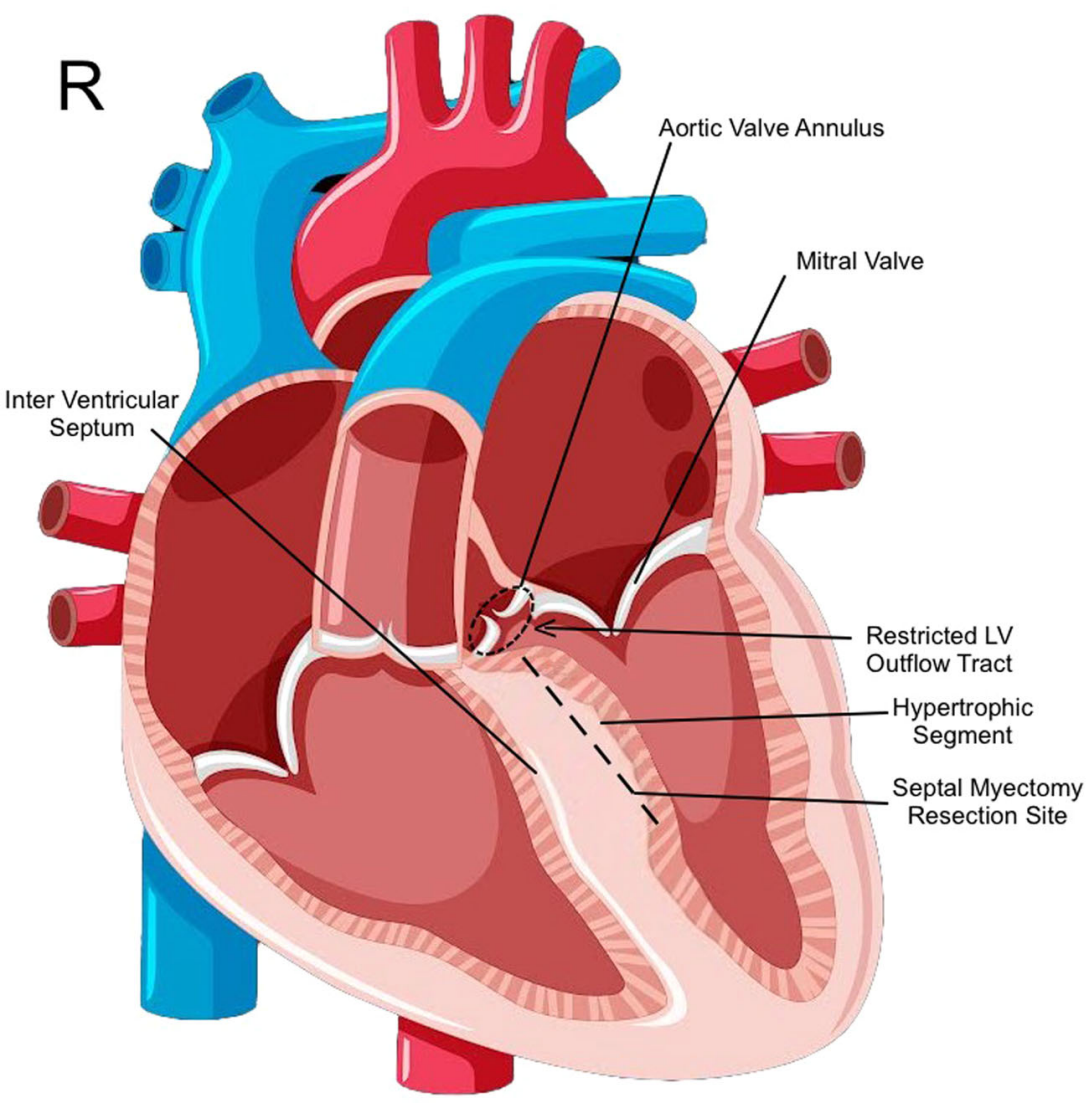
Schematic view of a heart with hypertrophic cardiomyopathy and site of septal myectomy.

Several medical treatments are available for HCM patients to reduce the symptoms. Furthermore, implantable cardioverter defibrillator (ICD) implantation might be indicated in some patients at risk of lethal arrhythmia. However, surgical treatment is gold‐standard in patients with severe and refractory HCM. Surgical septal myomectomy consisted of partial resection of hypertrophied interventricular septum (IVS) under CPB and was first introduced in 1960 by Morro et al.^9^ (Figure 1). An extended version of this surgery was further developed, which involved extended septal resection accompanied by releasing the abnormal septum‐free wall and the papillary muscle attachments.^10^

Moreover, due to mitral valve (MV) abnormalities in HCM patients and further relief of LVOT obstruction, mitral valve replacement (MVR) or repair (MVr) is necessary for some of these patients.^11^, ^12^, ^13^ Therefore, surgeons may perform septal myectomy and MVR simultaneously to improve symptoms for HCM patients.^14^ However, there is a lack of clinical evidence comparing these treatments directly. As a result, the HCM team at each medical facility ultimately decides which treatment to use.^15^, ^16^ Moreover, little has been reported on the long‐term outcomes of HCM patients undergoing surgical treatment in Iran.

In this study, we aimed to report long‐term outcomes of HCM patients undergoing septal myectomy or MVR in a single Iranian center and compare the results between different types of surgeries.

## 2 METHODS

### 2.1 Study design and setting

We conducted a retrospective cohort study on HCM patients undergoing surgical treatment. Surgical treatment was indicated for HCM patients with severe refractory symptoms who were unresponsive to medical treatment. Interventions were conducted at Tehran Heart Center, a referral heart center in the capital of Iran. HCM patients referred to our center are evaluated by an HCM team consisting of adult cardiologists, cardiovascular imaging specialists, interventional cardiologists, and cardiothoracic surgeons. The HCM team designed the treatment plan for each patient according to multimodality imaging studies and the latest guidelines. The National Ethics Committee approved the study. Informed consent was obtained from all patients for an anonymous report of their data in the study. (Ethics Approval Code: IR.AJAUMS.REC.1401.168).

### 2.2 Participants

All patients diagnosed with HCM and underwent septal myectomy, MVR, or both procedures were included in the study. The study period was between 2005 and 2021. Patients were divided into three groups according to their type of surgery, including septal myectomy, MVR, or a combination of both surgeries. Results were compared between these groups. Patient follow‐up data were acquired according to hospital and clinic records and contacting the patients.

Surgical intervention was indicated for patients according to the latest American Heart Association guidelines and consultations with the HCM team during referrals. Selected patients for surgical intervention had the following criteria.

1. Severe and refractory heart failure symptoms, such as dyspnea, fatigue, and fluid retention, significantly affected the patient's quality of life despite optimal medical therapy.
2. LVOT obstruction with a resting or provocable LVOT gradient ≥50 mmHg, as measured by transthoracic echocardiography.
3. Patients with high‐risk features, such as documented ventricular tachycardia or syncope, which require ICD placement combined with surgical septal myectomy in select cases, according to the decision of the HCM team.
4. Patients with a family history of sudden cardiac death or HCM‐related complications, which required prophylactic ICD placement and surgical septal myectomy to reduce the risk of adverse outcomes.

The selection of surgical approach for each patient was individually determined by the treatment team, taking into consideration both the unique patient characteristics and the echocardiographic profile of the hypertrophic segment. In cases where septal hypertrophy predominantly affected the LVOT, restricting the blood flow out of the left ventricle, a septal myectomy procedure was conducted. For patients presenting with an intrinsic pathology of the MV or exhibiting significant MR attributed to SAM or degenerative mitral alterations, concurrent MVR was performed alongside septal myectomy, especially when there was an expectation of sustained issues despite myectomy. Alternatively, MVR surgery in the absence of concomitant septal myectomy was chosen for patients with a relatively lesser thickness of the IVS in the presence of substantial MV malfunctions.

### 2.3 Variable and measurements

Patient medical records were reviewed, and data were collected on patient demographics, comorbidities, history, physical examination, echocardiography, electrocardiograms, surgical characteristics, in‐hospital complications, and long‐term outcomes. The collected data were entered into a database for analysis.

Experienced cardiologists performed all baseline and follow‐up echocardiographic assessments using commercially available ultrasound machines (Vivid S60 and [GE Healthcare] and Philips Affiniti [Koninklijke Philips N.V]). Patients were diagnosed with HCM if they had IVS thickness of more than 15 mm in one or more ventricle segments, not explained by alternative conditions such as hypertension and other valvular or congenital malformations.^17^ The LVOT gradient was calculated by using the modified continuous‐wave Doppler and Bernoulli equation. Systolic anterior motion (SAM) was detected as an early systolic movement of the MV leaflets toward the IVS. The severity of SAM was reported according to the MV motion detected by echocardiograms considering the resultant LVOT gradient and MR.

Long‐term cardiac implantable electronic device (CIED) implantation was defined as the placement of an ICD, permanent pacemaker (PPM), or cardiac resynchronization therapy device (CRT).

Long‐term major adverse cardiac and cerebrovascular event (MACCE) was defined as a composite endpoint measuring the occurrence of significant adverse cardiovascular and cerebrovascular outcomes, including mortality, myocardial infarction, stroke, and the need for second cardiovascular interventions, such as cardiac surgeries or percutaneous interventions.

### 2.4 Surgical procedure

**Figure 2:**
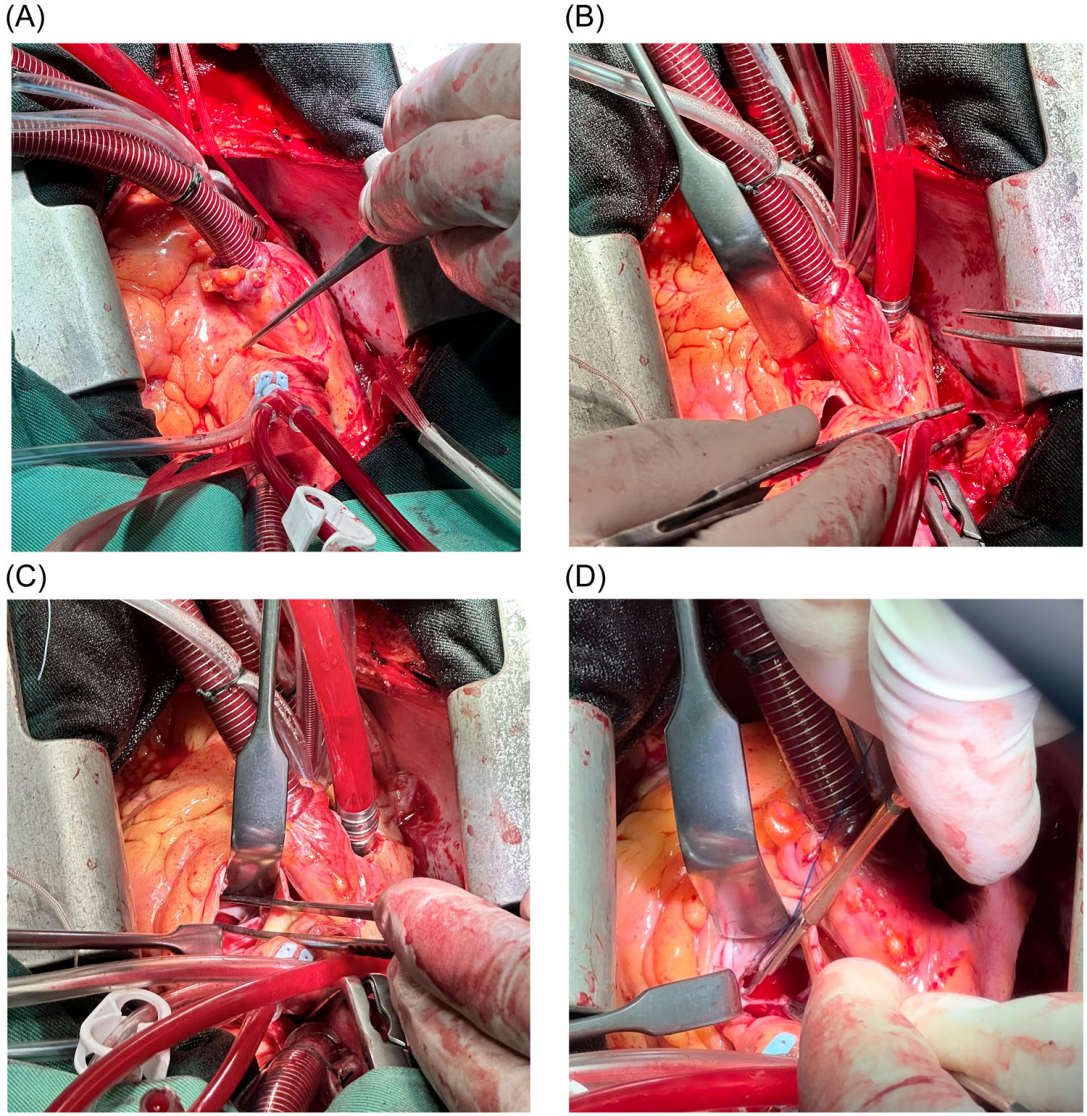
Surgical view of septal myectomy procedure via aortic approach with extended aortotomy (A and B). Exposure and subsequent resection of the hypertrophied septum (C and D).

The myectomy surgeries were performed under a cardiopulmonary bypass (CPB) utilizing a two‐staged cannula for standard central aortic and right atrial appendage cannulation. The transaortic method for septal myectomy was employed, which involved an S‐shaped Aortotomy extended towards the center of the noncoronary cusp.^18^ The LVOT was evaluated by gently applying a wide‐angle Langenbeck retractor over the right coronary cusp.^18^ A traction suture was typically employed on the ventricular septum to facilitate the exposure and manipulation of the specific area of the myocardium that necessitates resection. Subsequently, septal resection was conducted using a 10‐blade surgical knife beginning below the nadir of the right aortic cusp, lateral to the membranous septum (Figures 1 and 2). The septal myectomy continued with an up‐and‐down movement of the surgical blade in a counterclockwise route toward the commissure between the left and right aortic cusps, extending toward the papillary muscles.^10^ The white fibrotic scar on the involved septum typically served as a marker for the extension of myectomy if present.^19^ The depth of myectomy was determined based on echocardiographic and surgical findings and the surgeon's experience. The remnant of the hypertrophied septum is precisely resected and trimmed with surgical scissors and forceps (Figures 1 and 2).

Furthermore, MVR was conducted using a left atrial approach for HCM patients with significant MR due to SAM or degenerative mitral changes. CPB for these patients was accomplished by utilizing a singular‐stage cannula configuration within the superior and inferior vena cava, enhancing the visualization of the MV and the left atrium. The MV was visualized by retracting the left atrium and was excised via an extensive incision along the annulus, extending from the posterior aspect to the superior vena cava, encompassing the apex of the left atrium. The prosthetic valve was then implanted in the MV position. Low‐profile mechanical valves were used for these patients to prevent future obstructions. The valve was secured to the annulus using sutures or an annuloplasty ring. Partial rings were preferred over complete rings for the annuloplasty to avoid a residual LVOTO. Moreover, the chordae tendineae, papillary muscles, and the anterior leaflet were severed to provide more ventricular space for the patients.^20^ Other concomitant procedures were performed as necessary, such as aortic or tricuspid valve repairs or replacement and coronary artery bypass grafting.

Transesophageal echocardiography was employed during the surgery to assess the success of septal myectomy and identify residual pathologies such as residual LVOT obstruction, MR, SAM, and potential complications such as ventricular septal defects or suspected residual transaortic gradients.

### 2.5 Follow‐up

Patients were scheduled for regular follow‐up visits after discharge, with a 1‐month follow‐up after the surgery and 6‐month follow‐up intervals afterward. Postoperative echocardiography was conducted at least a week after the surgery after achieving a stable hemodynamic condition. Additional echocardiography follow‐ups were performed in subsequent visits if required by the treatment team.

### 2.6 Statistical analysis

Continuous variables were expressed as mean ± standard deviation for variables with a normal distribution or median and quartiles for variables without a normal distribution. Categorical variables were presented as frequencies and percentages. ANOVA (analysis of variance) was used to test for significant differences in the mean values of continuous variables with a normal distribution across the three groups. The Kruskall‐Wallis test was used to compare continuous variables without a normal distribution. Pearson chi‐square test and Fisher's exact test compared categorical variables between groups according to the expected cell counts. Preoperative and postoperative echocardiographic data were compared using paired sample *T* test and Wilcoxon test according to the distribution of variables. Kaplan–Meier analysis was used to estimate the survival, MACCE, and CIED emplacement rate. Post hoc tests were conducted to determine which treatment groups differed significantly. A *p* value of less than 0.05 was considered statistically significant. Statistical analyses were performed using SPSS Statistics for Windows, version 23.0 (IBM Corp.) and Stata Statistical Software, release 15.2 (Stata Corp LLC.)

## 3 RESULTS

### 3.1 Baseline characteristics

All of the HCM patients who underwent septal myectomy or MVR at Tehran Heart Center between the years 2005 and 2021 were included in the study, resulting in a total of 102 patients. Twenty‐six patients underwent septal myectomy, 23 had an MVR surgery, and 53 patients had a combination of both procedures (septal myectomy + MVR). Baseline patient characteristics are reported in Table 1. The mean age of the patients was 53.3 ± 16.9 years, and the majority were male (56%). Patients were divided into three groups according to their type of surgery (septal myectomy, MVR, myectomy, and MVR).

**Table 1:** Characteristics of hypertrophic cardiomyopathy patients who underwent myectomy, mitral valve replacement (MVR), or both.

Patient characteristics			Total (*n* = 102)	Myectomy (*n* = 26)	MVR (*n* = 23)	Myectomy + MVR (*n* = 53)	*p* Value
Age			53.3 ± 16.9	48.7 ± 20.6	57.9 ± 13.7	53.3 ± 16.9	0.162
Male sex			57 (56%)	12 (46.2%)	14 (60.9%)	31 (58.5%)	0.50
BMI			27.5 ± 5.6	26.8 ± 5.6	26.2 ± 7.1	28.4 ± 4.7	0.21
Diabetes			10 (9.8%)	1 (3.8%)	5 (21.7%)	4 (7.5%)	0.12
Dislipidemia			39 (38.2%)	6 (23.1%)	9 (39.1%)	24 (45.3%)	0.16
Hypertension			32 (31.4%)	8 (30.8%)	9 (39.1%)	15 (28.3%)	0.64
Family history of CVD			27 (26.5%)	7 (26.9%)	6 (26.1%)	14 (26.4%)	0.99
Addiction							
Opium, *n*/*N* (%)			3 (2.9%)	1 (3.8%)	1 (4.3%)	1 (1.9%)	0.79
Smoking, *n*/*N* (%)			26 (25.5%)	6 (23.1%)	12 (52.2%)	13 (24.5%)	0.82
Past medical history							
CVA			4 (3.9%)	1 (3.8%)	0 (0%)	3 (5.7%)	0.80
MI			12 (11.8%)	4 (15.4%)	1 (4.3%)	7 (13.2%)	0.51
Previous cardiac interventions							0.94
PCI			3 (2.9%)	1 (3.8%)	0 (0%)	2 (3.8%)	
Cardiac surgery			6 (5.9)	1 (3.8%)	2 (8.7%)	3 (5.7%)	
NYHA classification							0.19
I			24 (23.5%)	8 (30.8%)	4 (17.4%)	12 (22.6%)	
II			52 (50.9%)	10 (38.5%)	13 (56.5%)	29 (54.7%)	
III			24 (23.5%)	8 (30.8%)	4 (17.4%)	12 (22.6%)	
IV			2 (2%)	0 (0%)	2 (8.7%)	0 (0%)	
Angina			61 (59.8%)	13 (50%)	14 (60.9%)	29 (54.7%)	0.28
Abnormal ECG			67 (65.7%)	10 (38.5%)	18 (78.3%)	39 (73.6%)	0.002
Atrial fibrilation			9 (8.8%)	1 (3.8%)	2 (8.7%)	6 (11.3%)	0.59
Heart block			14 (13.7%)	4 (15.4%)	2 (8.7%)	8 (15.1%)	0.99
Q wave			13 (12.7%)	2 (7.7%)	1 (4.3%)	10 (18.9%)	0.32
ST depression			9 (8.8%)	5 (19.2%)	1 (4.3%)	3 (5.7%)	0.71
T wave inversion			49 (48.0%)	8 (30.8%)	12 (52.2%)	29 (54.7%)	0.13
Medication							
Β‐blockers			79 (77.5)	20 (76.9%)	17 (73.9%)	42 (79.2%)	0.87
ACE‐inhibitors			14 (13.7%)	4 (15.4%)	7 (30.4%)	3 (5.7%)	0.21
Nitrates			16 (15.7%)	3 (11.5%)	6 (26.1%)	7 (13.2%)	0.45
Anticoagulants			12 (11.8%)	1 (3.8%)	8 (34.8%)	3 (5.7%)	0.002
Diuretics			22 (21.6%)	1 (3.8%)	10 (43.5%)	11 (20.8%)	0.008
Asprin			32 (31.4)	8 (30.8%)	8 (34.8%)	16 (30.2%)	0.87
Lab tests							
Hb			13.7 ± 1.9	13.9 ± 1.7	13.3 ± 1.9	13.8 ± 2.0	0.45
FBS			88 (78–97)	88 (78–91)	93 (82–110)	87 (78–94)	0.30
Cr			1 (0.8–1.2)	1.0 (0.8–1.3)	1.1 (0.9–1.2)	0.9 (0.8–1.1)	0.035
TG			133 (105–181)	127.5 (87–165)	113.5 (97–133)	159.0 (118–192)	0.02
LDL			109 (85–128)	106 (83–132)	102 (82–129)	110 (87–129)	0.90
HDL			41 (32.25–47)	43 (38–46)	41.0 (34–48)	38 (31–47)	0.17
Total cholesterol			170 (143–200)	170 (139–205)	163 (140–192)	175 (149–199)	0.90
Surgical characteristics							
Concomitant surgeries			42 (41.1%)	12 (11.8%)	9 (8.8%)	21 (20.6%)	0.84
CABG			20 (19.6%)	2 (7.7%)	6 (26.1%)	12 (22.6%)	
AVR			14 (13.7%)	8 (30.8%)	1 (4.3%)	5 (9.4%)	
TVR			6 (5.9%)	0	1 (1%)	5 (4.9%)	
PFO closure			1 (1%)	1 (1%)	0	0	
VSD closure			1 (1%)	0	1 (1%)	0	
Operative characteristics							
Perfusion time (min)			111 (77–151)	95 (69–140.5)	93 (77–122)	125 (79–163)	0.15
Cross clamp time			69.5 (49–96)	55 (35–79)	60 (36.5–83)	101.5 (80–118)	0.16
Ventilation time (h)			9 (6–14)	9 (6.5–11)	12.5 (7–18)	9 (5–14.5)	0.59
ICU time (h)			39 (11–71)	19 (9.5–47)	44.5 (13–79)	43 (14–71.5)	0.80
IABP			5 (4.9%)	1 (3.8%)	1 (4.3%)	3 (5.7%)	0.91
Inotropes			51 (50%)	15 (57.7%)	14 (60.9%)	22 (41.5%)	0.09
Length of stay			9 (7–16)	11 (7–15)	9 (8–13)	9 (7–14)	0.90
In‐hospital complications			12 (11.8%)	1 (3.8%)	5 (21.7%)	6 (11.3%)	0.17
CVA‐TIA			2 (2%)	0 (0%)	2 (8.7%)	0 (0%)	
Renal failure			5 (4.9%)	1 (3.8%)	1 (4.3%)	3 (5.7%)	
Infection			3 (2.9%)	0 (0%)	1 (4.3%)	2 (3.8%)	
Tamponade			2 (2%)	0 (0%)	1 (4.3%)	1 (1.9%)	
Heart block			6 (5.9%)	3 (11.5%)	1 (4.3%)	2 (3.8%)	0.47
Blood transfusion			37 (36.3%)	9 (34.6%)	5 (21.7%)	23 (43.4%)	0.19
Pleural/pericardial effusion			13 (12.7%)	1 (18.9%)	2 (4.3%)	10 (17.7%)	0.19
Pericardial effusion			8 (7.8%)	1 (3.8%)	1 (4.3%)	6 (11.3%)	
Pleural effusion			7 (6.9%)	1 (3.8%)	1 (4.3%)	5 (9.4%)	

Abbreviations: AVR, aortic valve repair or replacement; BMI, body mass index; CVA, cerebrovascular accident; CVD, cardiovascular disease; ECG, electrocardiogram; FBS, fasting blood sugar; Hb, hemoglobin; HDL, high‐density lipoprotein; LDL, low‐density lipoprotein; MI, myocardial infarction; NYHA, New York Heart Association; PCI, percutaneous coronary intervention; TG, triglyceride.

The prevalence of comorbidities among the patients was as follows: 9.8% had diabetes, 38.2% had dyslipidemia, 31.4% had hypertension, and 2% had chronic obstructive pulmonary disease (COPD). 26.5% of the patients had a family history of cardiovascular diseases (CVD), only 2.9% were using opium, and 25.4% were smokers. Past medical history of the patients included 3.9% cerebral vascular accident (CVA), 1% renal failure (RF), and 11.8% myocardial infarction (MI). Moreover, nine (8.8%) had previous cardiac interventions, including percutaneous coronary intervention in three (2.9%) and cardiac surgery in six (5.8%) patients. There was no significant difference among the three groups regarding these characteristics.

Most (76.5%) patients were in NYHA class II or higher. 59.8% of the patients had angina, and 65.7% had an abnormal ECG. The prevalence of abnormal ECG was significantly lower in the septal myectomy group (38.5%) compared to the other two groups (78.3% and 73.6%, *p* value = 0.002). The most common medication used was β‐blockers (77.5%), similar in all three groups.

### 3.2 Surgical characteristics

Surgical characteristics are reported in Table 1. The mean perfusion time was 111 min (77–151), and the mean cross‐clamp time was 69.5 min (49–96), which were relatively higher in the MVR + Myectomy groups although not statistically significant (*p* values = 0.15 and 0.16). The median ventilation time and ICU stay were 9 (6–14) and 39 h (11–71), indicating no statistically significant difference among the three groups (*p* values = 0.59 and 0.80). Inotropes were used in 50% of the patients, and 5 (4.9%) required intra‐aortic balloon pump support. Concomitant surgeries were conducted in 42 (41%) patients, with the most common surgery being CABG (20 patients), followed by Aortic valve repair (7 patients) or replacement (9 patients) and Tricuspid valve repair (4 patients) or replacement (2 patients). A concomitant patent foramen ovale closure was performed in an HCM patient with a significant SAM who underwent MVR. A ventricular septal defect patch closure, was performed in one HCM patient with a midventricular hypertrophy and obstruction subsequent to septal myectomy.

### 3.3 Clinical outcomes

The median hospital stay was 9 days (7–16) without a significant difference between the three groups (*p* values = 0.90). In‐hospital complications were reported in 12 (11.8%) of the patients, including cerebrovascular accident or transient ischemic attack (TIA) (2%), RF (4.9%), infection (2.9%), and tamponade (2%). These complications were relatively lower in the septal myectomy group (3.8%) compared to other groups (21.7% and 11.3%); however, the difference was not statistically significant (*p* value = 0.17). Blood transfusion was required for 37 (36.3%) patients, and 6 (5.9%) patients were diagnosed with a heart block (5.9%), which was relatively higher in the septal myectomy group although not statistically significant (11.5% vs. 4.3% and 3.8%, *p* value = 0.47). Out of six patients with heart block, four underwent ICD implantation, and two were managed conservatively.

### 3.4 Echocardiographic outcomes

The preoperative and postoperative echocardiographic characteristics are shown in Table 2 and Supporting Information: Table S1. Mild, moderate, and severe preoperative SAM was reported in 7.8%, 37.3%, and 48% of the patients. Only 7.9% of the patients had remaining SAM after the surgery, which was mild in 6.9% and severe in one case. 98% of the patients had a detectable preoperative MR, which was severe in 51% and moderate in 21.6%, while only 7.8% had moderate or severe postoperative MR.

**Table 2:** Comparison of echocardiographic characteristics of patients with hypertrophic cardiomyopathy undergoing myectomy, mitral valve replacement (MVR), or both.

Surgery_type	Total	Myectomy	MVR	Myectomy + MVR	*p* Value
Preoperative					
Preoperative IVS d	21.4 ± 5.8	21.9 ± 7.3	18.6 ± 4.9	22.4 ± 4.9	0.025
Preoperative LVOTMG	35 (15–59)	35 (10–49)	10 (3–47)	35 (23–58)	0.082
Preoperative LVOTPG	70 (37–101)	70 (33–100)	17 (7–76)	71 (54–107)	0.016
Postoperative					
Postoperative IVS d	16.5 ± 5.3	15.1 ± 5.1	15.7 ± 4.6	17.5 ± 5.5	0.13
Postoperative LVOTMG	5 (4–9)	6 (3–11)	4 (4–7)	5 (4–9)	0.31
Postoperative LVOTPG	10 (8–15)	11 (7–22)	9 (7–13)	10 (8–15)	0.14

Abbreviations: IVS d, interventricular septum thickness in diastole; LVOTMG, left ventricular outflow tract mean gradient; LVOTPG, left ventricular outflow tract peak gradient; myectomy, septal myectomy.

The global ejection fraction (EF) decreased from 58.1 ± 7 to 55 ± 6.2 after the surgery (*p* = 0.001). The IVS thickness significantly reduced from 21.4 ± 5.8 to 16.5 ± 5.3 (*p* = 0.001) after the surgery. The LVOT peak and mean pressure gradients decreased considerably from 70 (37–101) and 35 (15–59) to 10 (8–15) and 5 (4–9) (*p* values = 0.001) after surgery.

A comparison of preoperative and postoperative echocardiographic results between the three groups is demonstrated in Table 2 and Supporting Information: Table S2. The MVR group had significantly lower preoperative IVS and LVOT PG (*p* Values = 0.015 and 0.008) compared to the other groups. Postoperative echocardiographic measurements did not indicate a statistically significant difference among the three groups other than aortic root and left atrium diameter, which was significantly higher in the MVR group compared to the other groups (*p* Values = 0.026 and 0.023). All three groups had significant reductions in IVS (*p* Values = 0.001, 0.031, and 0.001), LVOT PG (*p* values = 0.001, 0.009, and 0.001), and LVOT mean gradient (*p* values = 0.001, 0.007, and 0.001). MR and SAM were also significantly reduced in all three groups (All *p* values = 0.001).

### 3.5 Long‐term outcomes

The median patient follow‐up time was 6.8 (4.9 – 8.7) years. The long‐term outcomes of the patients are reported in Table 3. The long‐term adverse cardiac events (MACCE) rate was 16.7%, and the first MACCE included acute coronary syndrome (2%), CVA (2%), all‐cause death (11.8%), and redo surgery (1%).

**Table 3:** Comparison of long‐term outcomes of patients with hypertrophic cardiomyopathy undergoing septal myectomy, MVR, or both.

Patient characteristics	Total (*n* = 102)	Myectomy (*n* = 26)	MVR (*n* = 23)	Myectomy + MVR (*n* = 53)	*p* Value
10‐Year mortality	15 (14.7%)	0 (0%)	6 (26.1%)	9 (17%)	0.012
10‐Year CIED emplacement	25 (24.5%)	11 (42%)	1 (4.3%)	13 (24.5%)	0.069
10‐Year MACCE	19 (18.6%)	0	7 (30.4%)	12 (22.6%)	0.004

Abbreviations: CIED, cardioverter defibrillator implantation; MACCE, major adverse cardiovascular and cerebral event; myectomy, septal myectomy; MVR, mitral valve replacement.

All‐cause mortality rates were 2.9% for in‐hospital mortality, 3.9% for 1‐year mortality, 10.8% for 5‐year mortality, and 14.7% for 10‐year mortality. Causes of mortality included cardiac events (4.9%), CVA (2%), cancer (2%), infection (1%), surgical complications (2%), pulmonary thromboembolism (1%), and other causes (2%).

**Figure 3:**
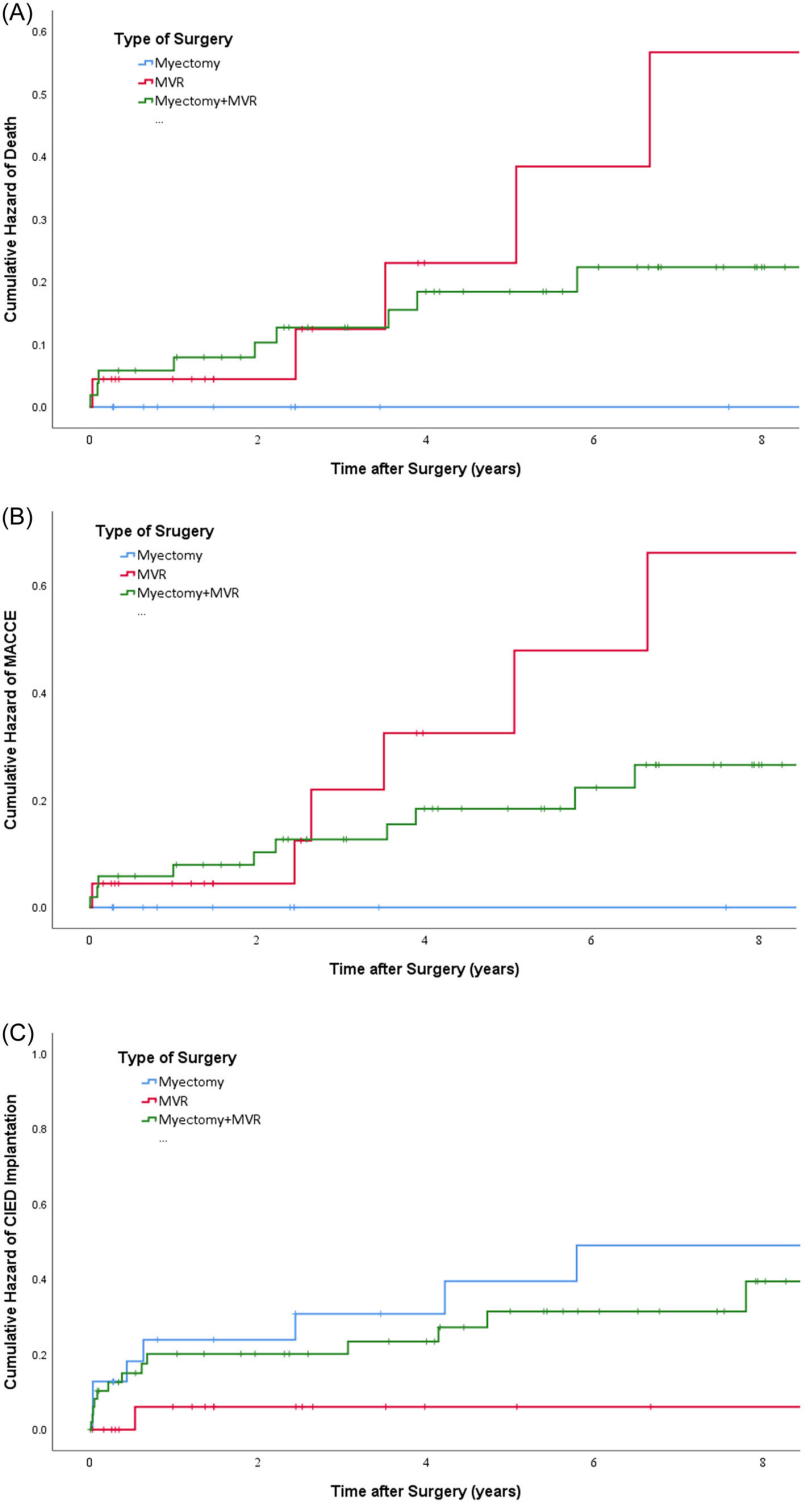
Comparison of Kaplan–Meier hazard curve for long‐term outcomes after surgery according to type of surgery. (A) Mortality, (B) MACCE, and (C) CIED implantation. CIED, cardioverter defibrillator implantation; MACCE, major adverse cardiovascular and cerebral event; myectomy, septal myectomy, MVR, mitral valve replacement.

A comparison of long‐term survival and MACCE in three surgery groups is presented in Figure 3. A statistically significant difference was reported in the long‐term outcomes of septal myectomy patients compared to other groups (*p* value = 0.012 for survival and 0.004 for MACCE). There were no reported MACCE or mortality in patients who underwent isolated septal myectomy surgery in the long‐term follow‐up.

Finally, the rate of CIED implantation during the follow‐up period was 5.9% for in‐hospital, 14.7% for 1 year, 19.6% for 5‐year, and 24.5% for 10‐year follow‐up. Survival analysis of three groups for long‐term CIED implantation is demonstrated in Figure 3. Long‐term CIED implantation was relatively lower in the patients who underwent isolated MVR compared to other groups (*p* value = 0.069).

## 4 DISCUSSION

HCM is a complex cardiac disorder characterized by various clinical symptoms, including reduced exercise tolerance, chest pain, dyspnea, and arrhythmias. After its introduction in 1960, septal myectomy has become the optimal intervention for HCM patients with refractory symptoms, positive SAM, and high LVOT gradient. It has been associated with effective outcomes with minimal complications. Furthermore, MVR surgery has also been implemented for these patients to correct MV malformations caused by this disorder^21^ and reduce LVOT gradient, resulting in decent symptom relief and acceptable complications.^22^, ^23^ The treatment team should carefully select the type of surgical treatment for each HCM patient, considering the patient's unique characteristics. The location of the hypertrophy is a crucial consideration when deciding on an intervention strategy. Septal myectomy is usually required when hypertrophy significantly impacts the LVOT. In contrast, MVR is indicated when the hypertrophic site is close to the MV and has compromised its functions or when the hypertrophic region is on the apical side of the septum and affects the papillary muscles.

Despite the widespread implementation of concomitant MVR with septal myectomy,^24^, ^25^, ^26^ recent studies suggest that avoiding MVR in cases where the MV abnormality is solely due to HCM can be associated with better outcomes.^27^, ^28^ Furthermore, alcohol septal ablation has recently become a promising surgical intervention alternative. However, septal myectomy remains the gold‐standard treatment for severe cases of HCM. This is due to a higher chance of requiring a pacemaker or re‐interventions after alcohol septal ablation.^29^, ^30^, ^31^

Most of the current data on these interventions are limited to developed countries, with inadequate data on the status of these interventions in the developing world. There are sparse data on the surgical treatment status for HCM patients in Iran. A recent study by Ghavidel et al. estimated that only 30 septal myectomy procedures are performed annually for Iranian HCM patients.^19^ This indicates that a large number of HCM patients who might benefit from surgical interventions are not receiving appropriate interventions. Several factors might contribute to the country's lower number of surgical interventions for HCM patients, including the limited number of cardiothoracic surgeons experienced in performing septal myectomy^32^ and the lack of sufficient data on long‐term outcomes interventions. Nonetheless, recent advancements in achieving a national registry for HCM patients and multidisciplinary HCM teams in Iranian heart centers can be beneficial in providing appropriate care for these patients.^33^ The long‐term outcomes of these registries can be essential in developing further interdisciplinary HCM teams across the country and achieving broader coverage for HCM patients.

The present study aimed to report and compare long‐term outcomes of HCM patients who underwent surgical treatment, including septal myectomy, MVR, or both procedures, in a single center in Iran over 17 years. The average age of our patients was similar to previous reports from Western countries.^28^, ^30^, ^34^ However, the average age in several other studies was relatively lower, possibly due to the inclusion of patients with congenital septal abnormalities.^19^, ^35^

Baseline echocardiographic characteristics of our patients indicated a significantly increased IVS and LVOT gradient in these patients (21.5 ± 5.8) accompanied by a normal EF in HCM patients. 71% of the patients had a detectable SAM before the surgery (65% had moderate or higher), and 72.6% of the patients had a moderate or higher MR, which was significantly corrected regardless of the type of surgery, except in one case where significant residual SAM was detected after the surgery. While patients receiving MVR are expected to have MR and SAM corrected, significant improvements have also been found in cases where an isolated septal myectomy surgery was undertaken. This shows that septal myectomy, regardless of the concomitant MVR operation, can indirectly improve MV function in HCM patients. This improvement can be attributed to several factors, including reduced backflow to the left atrium due to LVOT obstruction relief,^36^ improved coaptation of MV leaflets,^37^ which is disrupted in HCM patients due to asymmetric hypertrophy and LVOT obstruction, and reduced mechanical stress on the MV and its supporting structures.^38^

Moreover, postoperative echocardiograms indicated acceptable LVOT gradient and IVS reductions in all three groups. Although patients receiving a septal myectomy procedure had the most significant reduction in LVOT gradient, MVR was also associated with a relative decrease in LVOT gradient, which can be attributed to the Normalization of LV Geometry,^39^ improved blood flow dynamics,^40^ and a reduction in Valve‐Septum Contact.^6^

However, a reduction in EF was also observed, which was still in the range for a normal EF. Previous reports on echocardiographic outcomes of HCM surgeries have reported similar results.^34^, ^41^, ^42^

The most common postoperative complication was heart block, which was reported in 5.9% of the patients requiring CIED. Although lower rates of this complication have been reported by several studies,^30^, ^43^, ^44^, ^45^ the 2016 nationwide reports from US hospitals reported a 9%–14% requirement for ICD implantation in these patients.^46^

Long‐term assessments of CIED implantation indicated that patients who underwent isolated or concomitant septal myectomy with MVR had a higher risk of requiring ICD implantation in the long term than those who underwent MVR. Possible disturbances of the cardiac conductive system during the septal myectomy procedure can contribute to this increased risk. However, the more severe status of HCM in patients who received septal myectomy can also explain the higher need for ICD implantation in the long term. Although data in this regard is limited, previous studies have reported a reduced need for ICD implantation after septal myectomy and a lower rate of ICD discharge in patients who had received ICD.^47^

The 10‐year survival rate of our patients was 85.3%. Ommen et al. reported a similar 83% 10‐year survival rate for HCM patients who underwent surgical interventions.^35^ However, recent studies have reported a lower 10‐year mortality rate for septal myectomy.^30^ Comparing the long‐term survival of our patient, we interestingly detected no mortality or MACCE in the isolated septal myectomy patients, which was statistically significant compared to other patients. Septal myectomy has historically been associated with better outcomes in HCM patients compared to MVR or combined surgeries.^14^, ^22^ Recent studies have also reported better outcomes after septal myectomy without MVR, suggesting that isolated septal myectomy can sufficiently reverse MR in HCM patients without needing a concomitant MVR.^28^ MVR surgery burdens patients in the long term due to anticoagulation treatments, infections, thromboembolism, and possible durability problems.^48^, ^49^ Therefore, avoiding MVR in HCM patients can significantly improve their outcomes, and MVR should be reserved only for HCM patients with intrinsic valve abnormalities or substituted by MV repair instead. Nevertheless, it should be noted that septal myectomy has limitations that necessitate MVR in patients with extended hypertrophies, complex anatomies, previous cardiac surgeries, and involvement of MV structures.

## 5 LIMITATIONS

The study design was retrospective and non‐randomized, which may introduce selection bias. Second, the study was conducted in a single center, which may limit the generalizability of the findings to other centers or countries. Third, the sample size was relatively small, limiting the study's statistical power. We did not include HCM patients who underwent MV repair in this study due to the limited number of such cases during the study period. Finally, some data, such as the type of HCM mutation, were unavailable, which may affect the outcomes of the surgical procedures.

## 6 CONCLUSION

Both septal myectomy and MVR are treatment options for HCM patients with severe and refractory symptoms. However, septal myectomy without concomitant MVR was associated with significantly higher long‐term survival. Therefore, septal myectomy can sufficiently correct HCM‐originated MR, and MVR surgery should only be reserved for HCM patients with intrinsic valvular defects. Further larger prospective studies are required to confirm this study's findings and provide more robust evidence regarding the long‐term outcomes of the two surgical procedures in HCM patients.

## AUTHOR CONTRIBUTIONS

**Aryan Ayati**: Data curation; investigation; writing—original draft; writing—review and editing; formal analysis. **Mehran Khoshfetrat**: Conceptualization; validation. **Saeed Davoodi**: Investigation. **Seyed Hossein Ahmadi Tafti**: Conceptualization; supervision. **Reza Arefizadeh**: Supervision; conceptualization; writing—review and editing.

## CONFLICT OF INTEREST STATEMENT

The authors declare no conflict of interest.

## ETHICS STATEMENT

All methods were carried out in accordance with relevant guidelines and regulations. Informed consent was obtained from all subjects and/or their legal guardian(s) for anonymous data publication. The protocol of this study has been approved by the board of research and the committee of medical ethics at Aja University of Medical Sciences (code of ethics: IR.AJAUMS.REC.1401.168).

## TRANSPARENCY STATEMENT

The lead authors, Seyed Hossein Ahmadi Tafti and Reza Arefizadeh, affirm that this manuscript is an honest, accurate, and transparent account of the study being reported; that no important aspects of the study have been omitted; and that any discrepancies from the study as planned (and, if relevant, registered) have been explained.

## Supporting information

Supporting information.

## Data Availability

The data sets regarding the current study are available from the corresponding author upon reasonable request.
