# Supplementary material for: Comparing long‐term outcomes of septal myectomy and mitral valve replacement in hypertrophic cardiomyopathy patients: A retrospective cohort study in Iran
Source: Health Sci Rep. 2024 Apr 15;7(4):e2045. doi: 10.1002/hsr2.2045 (PMC11019255; doi:10.1002/hsr2.2045)
Supplement: Supplementary file 1 — Supporting information. [file HSR2-7-e2045-s001.docx]

**Table S1** Echocardiographic characteristics of patients with hypertrophic cardiomyopathy before and after surgery

|  | **Pre Operative** | | **Post Operative** | | **P-Value** | |
| --- | --- | --- | --- | --- | --- | --- |
| EF | 58.1▒±▒7 | | 55▒±▒6.2 | | .000 | |
| IVS d | 21.4▒±▒5.8 | | 16.5▒±▒5.3 | | .000 | |
| Aortic Root | 30.6▒±▒4.6 | | 30.4▒±▒4.4 | | .350 | |
| left Atrium | 44.7▒±▒9.8 | | 44.2▒±▒10.8 | | .395 | |
| LVD d | 44.5▒±▒7 | | 44.4▒±▒6.4 | | .989 | |
| LVD s | 27.5▒±▒7 | | 29.4▒±▒6.5 | | .016 | |
| PW d | 11.4▒±▒3 | | 11.8▒±▒2.7 | | .287 | |
| RVD d | 28.6▒±▒4.3 | | 28.9▒±▒3.5 | | .432 | |
| LVOTMG | 35(15-59) | | 5(4-9) | | 0.001 | |
| LVOTPG | 70(37-101) | | 10(8-15) | | 0.001 | |
| **SAM** |  | |  | | 0.001 | |
| None | 7 (6.9%) | | 94(92.2%) | |  |  |
| Mild | 8 (7.8%) | | 7(6.9%) | |  |  |
| Moderate | 38 (37.3%) | | 0(0%) | |  |  |
| Severe | 49 (48%) | | 1(1%) | |  |  |
| **MR** |  |  | | 0.001 | |  |
| None | 2(2%) | 54(52.9%) | |  |  |  |
| Mild | 26(25.5%) | 40(39.2%) | |  |  |  |
| Moderate | 22(21.6%) | 5(4.9%) | |  |  |  |
| Severe | 52(51%) | 3(2.9%) | |  |  |  |
| EF: Ejection Fraction, IVS d: Interventricular Septum Thickness during diastole, LVD d: Left Ventricular Diameter during diastole, LVD s: Left Ventricular Diameter during systole, PW d: Posterior Wall Thickness during diastole, RVD d: Right Ventricular Diameter during diastole, LVOTMG: Left Ventricular Outflow Tract Mean Gradient, LVOTPG: Left Ventricular Outflow Tract Peak Gradient, SAM: Systolic Anterior Motion of Mitral Valve, MR: Mitral Regurgitation. | | | | | |  |

Table S2 Comparison of Echocardiographic characteristics of patients with hypertrophic cardiomyopathy undergoing Myectomy, Mitral Valve Replacement, or both

| **Surgery_Type** | **Total** | **Myectomy** | **MVR** | Myectomy+MVR | P-Value |
| --- | --- | --- | --- | --- | --- |
| **Preoperative** |  |  |  |  |  |
| Preoperative EF | 58.1▒±▒7 | 58.8▒±▒6 | 55.6▒±▒9.6 | 58.8▒±▒5.9 | 0.15 |
| Preoperative IVS d | 21.4▒±▒5.8 | 21.9▒±▒7.3 | 18.6▒±▒4.9 | 22.4▒±▒4.9 | 0.025 |
| Preoperative Aortic Root | 30.6▒±▒4.6 | 29.3▒±▒5.2 | 31.7▒±▒4.8 | 30.9▒±▒4.2 | 0.17 |
| Preoperative left Atrium | 44.7▒±▒9.8 | 41.6▒±▒6.1 | 47.1▒±▒7.3 | 45.2▒±▒11.7 | 0.13 |
| Preoperative LVD d | 44.5▒±▒7 | 42.2▒±▒6.5 | 48.9▒±▒8.2 | 43.6▒±▒5.8 | 0.001 |
| Preoperative LVD s | 27.5▒±▒7 | 25.3▒±▒6.6 | 31.4▒±▒8.4 | 26.8▒±▒5.9 | 0.005 |
| Preoperative PW d | 11.4▒±▒3 | 11.2▒±▒1.9 | 10.9▒±▒2.8 | 11.8▒±▒3.4 | 0.43 |
| Preoperative RVD d | 28.6▒±▒4.3 | 27.3▒±▒3.5 | 29.9▒±▒4.1 | 28.6▒±▒4.5 | 0.12 |
| Preoperative LVOTMG | 35(15-59) | 35(10-49) | 10(3-47) | 35(23-58) | 0.082 |
| Preoperative LVOTPG | 70(37-101) | 70(33-100) | 17(7-76) | 71(54-107) | 0.016 |
| **Postoperative** |  |  |  |  |  |
| Postoperative EF | 55▒±▒6.2 | 56.1▒±▒7.1 | 53.2▒±▒6.7 | 55.3▒±▒5.4 | 0.26 |
| Postoperative IVS d | 16.5▒±▒5.3 | 15.1▒±▒5.1 | 15.7▒±▒4.6 | 17.5▒±▒5.5 | 0.13 |
| Postoperative Aortic Root | 30.4▒±▒4.4 | 30.1▒±▒4.5 | 32.6▒±▒4.5 | 29.6▒±▒4.2 | 0.026 |
| Postoperative left Atrium | 44.2▒±▒10.8 | 39.2▒±▒6.2 | 46.5▒±▒7.1 | 45.7▒±▒13 | 0.023 |
| Postoperative LVD d | 44.4▒±▒6.4 | 44.4▒±▒6.3 | 45.1▒±▒6.5 | 44.2▒±▒6.4 | 0.86 |
| Postoperative LVD s | 29.4▒±▒6.5 | 27.7▒±▒7.2 | 29.4▒±▒5.5 | 30.2▒±▒6.6 | 0.30 |
| Postoperative PW d | 11.8▒±▒2.7 | 11.8▒±▒3 | 12▒±▒2.6 | 11.7▒±▒2.7 | 0.90 |
| Postoperative RVD d | 28.9▒±▒3.5 | 28▒±▒3.9 | 29.6▒±▒4 | 28.9▒±▒3.1 | 0.32 |
| Postoperative LVOTMG | 5(4-9) | 6(3-11) | 4(4-7) | 5(4-9) | 0.31 |
| Postoperative LVOTPG | 10(8-15) | 11(7-22) | 9(7-13) | 10(8-15) | 0.14 |
| EF: Ejection Fraction; IVS d: Interventricular Septum thickness in diastole; LVD d: Left Ventricular Diameter in diastole; LVD s: Left Ventricular Diameter in systole; PW d: Posterior Wall thickness in diastole; RVD d: Right Ventricular Diameter in diastole; LVOTMG: Left Ventricular Outflow Tract Mean Gradient; LVOTPG: Left Ventricular Outflow Tract Peak Gradient; MVR: Mitral Valve Replacement; Myectomy: Septal Myectomy; | | | | | |
